# Current Antivirals and Novel Botanical Molecules Interfering With Herpes Simplex Virus Infection

**DOI:** 10.3389/fmicb.2020.00139

**Published:** 2020-02-11

**Authors:** Diana M. Álvarez, Estefanía Castillo, Luisa F. Duarte, José Arriagada, Nicolás Corrales, Mónica A. Farías, Adolfo Henríquez, Cristian Agurto-Muñoz, Pablo A. González

**Affiliations:** ^1^Millennium Institute on Immunology and Immunotherapy, Departamento de Genética Molecular y Microbiología, Facultad de Ciencias Biológicas, Pontificia Universidad Católica de Chile, Santiago, Chile; ^2^Centro de Biotecnología, Universidad de Concepción, Concepción, Chile; ^3^Departamento de Ciencia y Tecnología de Alimentos, Facultad de Farmacia, Universidad de Concepción, Concepción, Chile

**Keywords:** HSV-1, HSV-2, natural antiviral compounds, antiviral extracts, phytopharmaceuticals, therapy

## Abstract

Herpes simplex viruses type 1 (HSV-1) and type 2 (HSV-2) are highly prevalent within the human population and are characterized by lifelong infections and sporadic recurrences due to latent neuron infection. Upon reactivations, HSVs may manifest either, symptomatically or asymptomatically and be shed onto others through mucosae body fluids. Although, HSVs can produce severe disease in humans, such as life-threatening encephalitis and blindness, the most common symptoms are skin and mucosal lesions in the oro-facial and the genital areas. Nucleoside analogs with antiviral activity can prevent severe HSV infection, yet they are not very effective for treating skin manifestations produced by these viruses, as they only reduce in a few days at most the duration of lesions. Additionally, HSV variants that are resistant to these antivirals may arise, especially in immunosuppressed individuals. Thus, new antivirals that can reduce the severity and duration of these cutaneous manifestations would certainly be welcome. Here, we review currently available anti-herpetic therapies, novel molecules being assessed in clinical trials and new botanical compounds reported in the last 20 years with antiviral activities against HSVs that might represent future treatments against these viruses.

## Introduction

Herpes simplex virus type 1 (HSV-1) and herpes simplex virus type 2 virus (HSV-2) are viruses belonging to the *Herpesviridae* family, *Alphaherpesvirinae* subfamily and *Simplexvirus* genus. HSV-1 and HSV-2 belong to the same family and subfamily than varicella zoster virus (VZV), yet VZV belongs to the *Varicellovirus* genus (McGeoch, 2009; Kinchington et al., 2012; Ibáñez et al., 2018). HSV-1 and HSV-2 are highly prevalent in humans, with global infections ranging 70 and 10% of the world population, respectively (Smith and Robinson, 2002; Schillinger et al., 2004; Looker et al., 2008; Chayavichitsilp et al., 2009; Doi et al., 2009) (World Health Organization, Regional Estimates^1^.

HSV-1 and HSV-2 can cause severe disease in immunocompetent adults and newborns, such as life-threatening encephalitis with sequelae, despite antiviral treatment (Whitley et al., 2007; Xu et al., 2007; Dinh et al., 2008; Handel et al., 2011). These viruses can also produce eye infections leading to visual impairment: currently, HSV-1 is the main cause of infectious blindness in developed countries (Farooq and Shukla, 2012). However, the most common clinical manifestations associated to HSV-1 and HSV-2 infections are *herpes labialis* and *herpes genitalis* (Lafferty et al., 1987; Mertz et al., 1998; Kaye and Choudhary, 2006; Paz-Bailey et al., 2008; Farooq and Shukla, 2012), which are characterized by the appearance of vesicular ulcers in the oro-facial and genital areas that gradually dry out into crusts and may last up to 14 days during primary infections and approximately 10 days during recurrences if no treatment is undertaken (Arduino and Porter, 2007). Lesion development is sequential and begins with a prodrome displaying erythema, then papules emerge which may progress into vesicles that break up giving way to the formation of ulcers. Finally, these ulcers dry out forming scabs, which are accompanied by residual swelling and finally healing (Spruance et al., 1977; Corey et al., 1983; Fatahzadeh and Schwartz, 2007). The lesions contain high amounts of virions and infiltrating leukocytes and may be painful with a burning sensation, ultimately impacting the quality of life of the affected individuals (Spruance et al., 1977; Dreno et al., 2012).

Nevertheless, not all the individuals infected with HSV-1 and HSV-2 manifest symptoms. It is estimated that herpetic recurrences due to HSV occur within a wide range of frequencies, varying between 20–50% and 80–90% for HSV-1 and HSV-2 infections, respectively after primary infection (Mertz et al., 1992; Benedetti et al., 1994; Cowan et al., 1994; Fleming et al., 1997). This means that 50–80% and 10–20% of individuals with HSV-1 and HSV-2 infection, correspondingly will not show clinical symptoms of infection. Yet, it is important to note that these persons will nevertheless shed infectious viral particles from the mucosae, which could infect other individuals (Johnston and Corey, 2015; Ramchandani et al., 2017). On the other hand, up to one-third of the persons that have had clinical symptoms during primary infection show frequent reactivations, which occur on average six times a year (Benedetti et al., 1994). Overall, it is currently estimated that 10–25% of the individuals that are infected with HSV manifest disease symptoms, particularly skin lesions in various forms (*herpes labialis*, *herpes genitalis*, *eczema herpeticum, zosteriforme* herpes, etc.) (Mertz et al., 1992; Cowan et al., 1994; Fleming et al., 1997). Taking into consideration the numbers outlined above, approximately 16 and 5% of the world population will manifest herpetic lesions with HSV-1 and HSV-2, respectively. This extremely high percentage of individuals affected by symptomatic HSV infection is non-negligible and undoubtedly encompasses a significant number of persons that would like to have access to more effective solutions against these viruses.

The reactivation of HSV-1 and HSV-2 from infected individuals is associated with numerous factors, such as immune-related and physicochemical stimuli, such as UV radiation, as well as menstruation, stress and traumatic events, among others (Perna et al., 1987; Rand et al., 1990). Upon viral reactivation, virions travel in a retrograde manner from the cell body of infected neurons in the trigeminal ganglia (orofacial infection), or the dorsal root ganglia (genital-associated infection), to sites neighboring epithelial cells and fibroblasts, nearby the original site of infection, forming new lesions that will repeat the process of additional neuron infection (Stevens and Cook, 1971; Lafferty et al., 1987; Benedetti et al., 1994). Given this scenario, it seems important to block neuron infection by HSV-1 and HSV-2 during primary infection or to treat neurons in such a way that these viruses do not reactivate from these cells. However, a prophylactic approach for HSV-1 and HSV-2 is yet not available in the form of a vaccine (Kwant and Rosenthal, 2004; Belshe et al., 2012) and the effective eradication of these viruses from neurons requires further research (van Diemen et al., 2016; van Diemen and Lebbink, 2017; Chen et al., 2018).

At present, there are several commercially available antivirals to treat skin lesions caused by HSV-1 and HSV-2. However, such drugs are somewhat ineffective for this type of clinical manifestation, as they only shorten the recovery time of the lesions in 1–2 days in most cases (Evans et al., 2000; Leflore et al., 2000). For some individuals, the effectiveness of these treatments may be imperceptible [meta-analysis: Chen et al. (2016)].

On the other hand, approximately 3.5–10% of immunosuppressed individuals (e.g., transplanted persons, HIV-positive individuals, those undergoing pharmacological treatments to depress the immune system, among others) may develop HSV-1 and HSV-2 variants that are resistant to the most commonly used antivirals (Stranska et al., 2005; Ziyaeyan et al., 2007; Suazo et al., 2014; Lolis et al., 2016). Although second line antivirals exist for these drug-resistant isolates, such as for acyclovir-resistant variants, unfortunately most of these compounds elicit numerous adverse effects (discussed below) (Javaly et al., 1999). In immunocompetent individuals, drug-resistant variants such as acyclovir-resistant isolates may also occur, yet at a lower frequency (approximately 1% of cases) (Bacon et al., 2002; Stranska et al., 2005; Ziyaeyan et al., 2007). Although this number seems small, considering the significant number of individuals infected with HSV-1 and HSV-2, the figure is substantial.

Several natural products have shown antiviral effects against HSV-1 and HSV-2, such as extracts, fractionated compounds and isolated molecules originated from marine organisms, microorganisms, fungi, animals, algae and plants, among others (Hassan et al., 2015). Among these bioactive products there are marine-derived nucleosides, such as spongothymidine and spongouridin, which gave origin to the first nucleoside analog drugs approved to control HSV-1 and HSV-2 infection (Hassan et al., 2015). Additionally, many plants used in herbal medicine have been reported worldwide to have antiviral effects against HSV-1 and HSV-2 (Lipipun et al., 2003; Jadhav et al., 2012; Brand et al., 2016; Li et al., 2018). Interestingly, different types of natural compounds display antiherpetic activity, such as alkaloids (Souza et al., 2007), polysaccharides (Damonte et al., 2009) and proteins (Gu et al., 2007).

Here, we review and discuss current antivirals used against HSV-1 and HSV-2, novel antiviral molecules tested in clinical trials against these viruses, as well as new compounds of botanical origin that have emerged in the last 20 years to treat herpes simplex viruses.

## Current Drugs Used Against HSV-1 and HSV-2

At present, there are numerous antiviral drugs for the treatment of HSV infections. Some commonly known anti-herpetic drugs that are currently being used include acyclovir (ACV), penciclovir, and famciclovir, which inhibit HSV-1 and HSV-2 infection by interfering with the viral DNA polymerase and hence, viral genome replication (Kukhanova et al., 2014). These drugs are also used to treat other herpesvirus infections, such as VZV and cytomegalovirus (human herpesvirus 5, HHV-5, CMV). Although several of these drugs, which are described in more detail below, help reduce disease and minimize potential severe damage, as well as to limit the spread of these viruses onto other individuals, most of these compounds only modestly reduce cutaneous manifestations (lesions) caused by herpes simplex viruses, particularly when used in the form of creams that are applied topically to the skin (Lebrun-Vignes et al., 2007; Hammer et al., 2018).

### Acyclovir

Numerous antivirals approved for the treatment of HSV-1 and HSV-2 infections are acyclic nucleoside and nucleotide analogs that interfere with the elongation of viral genome during replication, which is carried out by the viral DNA polymerase (UL30). Acyclovir (ACV) is an acyclic guanosine analog discovered in the sponge *Cryptotethya crypta* and is at present the most frequently used compound to treat HSV-1 and HSV-2, mainly because of its low price, tolerability and safety (Hassan et al., 2015). Importantly, acyclovir needs to be activated intracellularly by its phosphorylation into acyclovir triphosphate for exerting its antiviral activity. This process is carried out by the viral thymidine kinase (TK, *UL23* gene), which catalyzes acyclovir into acyclovir monophosphate, thus increasing the concentration of acyclovir within infected cells by reducing its exit from the cell (Reusser, 1996; Kukhanova et al., 2014). Additional phosphorylations are carried out by cellular kinases and once in its triphosphate form, acyclovir becomes a substrate for the viral DNA polymerase interfering with DNA synthesis (Reusser, 1996; De Clercq, 2013). Importantly, inhibition of the synthesis of new viral genome copies, which translates lesser formation of novel infectious viral particles, does not affect latent virus within host neurons and hence, does not cure infection (Poole and James, 2018). Other limitations related to the treatment with ACV also exist. For instance, oral intake of ACV has an absorption efficiency of only 15–30% (Bean, 1992), and previous reports indicated that senior patients that had renal problems could experience significant neurotoxicity, because they were not able to properly excrete this drug (Chau, 2018). Another important limitation associated to ACV is the ability of HSV-1 and HSV-2 to mutate and generate variants that are resistant to this drug by acquiring point mutations in the gene encoding for the viral thymidine kinase (TK), which decreases enzyme expression or modifies substrate specificity abrogating acyclovir phosphorylation, or by acquiring mutations in the gene encoding for the viral DNA polymerase (*UL30*), which may enable HSV-1 and HSV-2 to replicate in the presence of ACV (Reusser, 1996). Such ACV-resistant variants occur mainly in immunosuppressed individuals, as they are otherwise generally attenuated in immunocompetent individuals (Field, 2001; Morfin and Thouvenot, 2003).

Oral intake of ACV for treating skin lesions produced by HSV-1 and HSV-2 only reduces the healing process in little more than 2 days (time to loss of scab), from 7.9 days (placebo group) to 5.8 days, if taken as soon as signs of the prodrome or erythema are detected (Spruance et al., 1990). Because these differences are moderate, although statistically significant, the clinical benefit of these antiviral drugs for the treatment of herpetic lesions has been somewhat questioned (Kroon, 1990; Mindel, 1991). Importantly, when the drug is taken at the stage of papule, a significant effect for ACV is not observed (Spruance, 1993). Furthermore, within this scenario, the recovery time of cutaneous lesions was found to be larger in the treated group (antiviral) than in the placebo group (8 days vs. 7.2 days, respectively). Regretfully, nearly 50% of patients fail to perceive initial stages of the prodrome and the erythema phases before papule formation and therefore these individuals will not be in time to start an effective oral treatment with ACV in such a way to significantly reduce the time of the herpetic lesions (Spruance et al., 1990; Spruance, 1993). Thus, treatment with ACV has poor benefits under these circumstances (Spruance et al., 1990).

As an alternative to oral intake, ACV can be applied topically as a cream. Although topical application of ACV over herpetic lesions at the papule stage has beneficial effects, these benefits are relatively weak, since they only reduce healing time in approximately one or two days during *herpes labialis* (Bean, 1992; Evans et al., 2000) and three days in genital herpes infections (Thin et al., 1983; Leflore et al., 2000).

### Valacyclovir, Penciclovir, and Famciclovir

Besides ACV, there are other alternatives to treat skin lesions caused by herpes simplex viruses, such as valacyclovir, penciclovir, and famciclovir, which are also considered first-line drugs to treat HSV-1 and HSV-2 and thus, are frequently used (Kimberlin and Whitley, 2007).

These drugs are nucleic acid analogs, similar to ACV with a shared mechanism of action that interferes with the function of the viral DNA polymerase (De Clercq, 2013). These compounds differ from each other mainly in their bioavailability, half-life in the body and dosing (Wagstaff and Bryson, 1994), yet similar to ACV they reduce herpetic lesions and associated pain in approximately 1–3 days, as compared to untreated groups when used topically (Emmert, 2000).

To increase the bioavailability of acyclovir, an L-valine ester of acyclovir was developed. Valacyclovir is a prodrug of ACV with enhanced absorption at the intestinal level (54%) (Soul-Lawton et al., 1995). Later, penciclovir was developed with the aim of being phosphorylated more rapidly than ACV, and consequently has a higher half-life than acyclovir (Hodge and Perkins, 1989). While acyclovir has a half-life of 0.7 h for HSV-1 and 1 h for HSV-2, penciclovir has a half-life of 10 h for HSV-1 and 20 h for HSV-2 (Luber and Flaherty, 1996). Famciclovir is a prodrug that derives into penciclovir and has increased oral bioavailability (Hodge et al., 1989). Clinical benefits granted by these drugs have also generated discussions on the recommendation of their use to treat skin lesions caused by herpes simplex virus infections (Chi et al., 2015; Chen et al., 2016).

Valacyclovir has also been approved for the treatment of HSV-1 and HSV-2 infections and clinical manifestations produced by HSV-1 and HSV-2, such as cold sores and recurrent genital herpes, as well as VZV and cytomegalovirus (Ormrod et al., 2000). Furthermore, famciclovir is approved for treating herpes viruses, such as HSV-1 and HSV-2 (genital herpes and orolabial herpes), as well as VZV (Simpson and Lyseng-Williamson, 2006).

Resistance to valacyclovir, penciclovir, and famciclovir can occur. Furthermore, there is cross-resistance between valacyclovir- and acyclovir-resistant HSV isolates (Reusser, 1996), because valacyclovir is derived from acyclovir (Moomaw et al., 2003). On the other hand, cross resistance to penciclovir and the prodrug famciclovir may arise in acyclovir-resistant HSV-1 isolates in immunocompromised patients (Boyd et al., 1993). Some studies have reported HSV-1 and HSV-2 resistance to penciclovir in cell cultures and in immunocompromised patients related to TK-deficiency (Boyd et al., 1993; Sarisky et al., 2002, 2003; Bacon et al., 2003).

### Ganciclovir, Cidofovir and Foscarnet

In the last decade, alternatives to ACV for herpesvirus treatment have emerged and become commercially available as therapeutic drugs. Ganciclovir is a nucleic acid analog that does not require viral proteins for its activation in the cell (i.e., viral TK) (Markham and Faulds, 1994; Prichard et al., 2011; Vere Hodge and Field, 2013; Poole and James, 2018). These compounds differ from each other either, in their molecular processing into an active form of an acyclic guanosine analog, or bioavailability which significantly determines the frequency of administration (De Clercq, 2013).

Ganciclovir is indicated for the treatment of CMV, particularly for systemic and ocular infections in immunosuppressed patients (Buhles et al., 1988; Poole and James, 2018). However, ganciclovir has also been reported to have antiviral activity against HSV-1 and HSV-2 and may be used for the treatment of herpetic keratitis (Smee et al., 1983; Matthews and Boehme, 1988; Chou and Hong, 2014). At present, there is an ongoing clinical study that is recruiting patients for assessing the effects of oral ganciclovir, together with femtosecond laser-assisted corneal debridement in the treatment of herpes simplex virus epithelial keratitis (ClinicalTrials.gov NCT03217474). Of note, ganciclovir has been reported to produce considerable adverse side effects in a high percentage of individuals (Kimberlin and Whitley, 2007), such as nephrotoxicity, neutropenia, myelosuppression, confusion, altered mental status, anxiety, ataxia, tremors, convulsions, fever, abnormal levels of liver enzymes in serum, diarrhea, and nausea, among others (Kimberlin and Whitley, 2007).

Other alternative drugs that have emerged in the last decades as second-line drugs that are commercially available are cidofovir (an acyclic nucleotide analog) (Ashley et al., 1988; Blot et al., 2000; James and Prichard, 2014) and foscarnet (a pyrophosphate analog) (Crumpacker, 1992; Wagstaff and Bryson, 1994). When compared to each other, these two compounds affect different steps in the viral replication cycle. Cidofovir acts as a nucleotide analog and polymerase inhibitor with a high affinity for the viral DNA polymerase, with cidofovir-diphosphate having 25–50 fold higher affinity for the viral DNA polymerase than the host DNA polymerase, causing thus a more effective block in the replication of viral DNA than acyclovir (Ho et al., 1991). Importantly, cidofovir has a phosphonate group that does not require an initial phosphorylation step by HSV proteins (Ho et al., 1991). Cidofovir has shown to be efficacious against acyclovir-resistant isolates of HSV-1 or HSV-2 *in vitro* (Chilukuri and Rosen, 2003). However, this drug has not been approved for the treatment of herpes simplex viruses in humans. Yet, a clinical study was carried out to evaluate the effectivity of topical cidofovir for refractory mucocutaneous HSV-1 and HSV-2 in AIDS; However, the results of this clinical study have not been reported (ClinicalTrials.gov Identifier: NCT00002116). On the other hand, foscarnet inhibits the viral DNA polymerase of herpesviruses by binding near to the pyrophosphate binding site that is needed for polymerase activity (Crumpacker, 1992; Poole and James, 2018). In contrast to nucleoside analogs, resistance to foscarnet only occurs because of mutations in the viral DNA polymerase gene (Reusser, 1996).

Regretfully, these two drugs also produce numerous adverse effects in patients, such as nephrotoxicity, azotemia, proteinuria, crystalluria, interstitial nephritis, acute tubular necrosis, increases in the concentrations of creatinine up to 50%, hypo- and hypercalcemia, hypo- and hyper-phosphatemia, and the formation of urogenital ulcers, among others (Deray et al., 1989; Sauerbrei, 2016). Because of these effects, it is recommended that patients receiving foscarnet be monitored clinically to control abnormalities in metabolites and electrolytes that may result in the alterations indicated above.

Resistance to foscarnet has been observed in immuno- compromised individuals, particularly in patients having undergone bone marrow transplants. On the other hand, there are only few studies reporting cidofovir-resistant HSV isolates. In one case, three patients with bone marrow transplants received cidofovir as a therapy, but nevertheless showed HSV-related diseases symptoms. Cidofovir resistance was confirmed in one of the three cases with the isolate showing mutations that truncated the C-terminal of the viral DNA polymerase (Wyles et al., 2005). Notably, cidofovir resistance has also been reported in children, particularly in three patients with hematopoietic stem cell transplants that received, in a prophylactic manner acyclovir and cidofovir together, because ganciclovir produced adverse effects. Unfortunately, these children showed HSV-related stomatitis during cidofovir treatment and the authors suggested that the treatment with cidofovir did not prevent HSV-1 reactivation in the patients (Dvorak et al., 2009). Nevertheless, cidofovir is considered a good option when encountering HSV isolates that express reduced amounts of enzymes that are related to the phosphorylation of nucleoside analogs, or resistance to foscarnet (Reusser, 1996). For example, Blot et al. (2000) reported a clinical case of a child affected by a variant of HSV-1 resistant to ACV and foscarnet (*in vitro*), in which case the treatment with cidofovir was effective against this drug-resistant HSV-1. Another study reporting an HSV-1 isolate resistant to both, ACV and foscarnet in a girl with lymphatic leukemia indicated that only cidofovir treatment was successful at helping avoid recurrent oral stomatitis (Bryant et al., 2001).

Due to the somewhat reduced clinical benefits of the drugs mentioned above in the treatment of skin lesions caused by herpes simplex viruses, new therapeutic alternatives have emerged in the most recent years. One of these alternatives is a combination of acyclovir and hydrocortisone for topical use (Xerese^®^, Medivir). This formulation reduces the duration of herpetic lesions by 1.6 days (compared to 1.0 days by acyclovir when applied alone as a topic in that study) and reduces the size of the lesion area by 50% (Strand et al., 2012). While this approach yields a statistically significant improvement in the treatment of herpetic lesions, it still evidences the need for identifying new drugs or drug combinations that have even better effectiveness.

Another relatively new drug to treat skin lesions caused by HSV-1 is docosanol 10% formulated as a topical cream (Abreva^®^, Avanir), which is the only FDA approved formulation available over the counter (OTC) to treat HSV-1 symptoms. This drug consists of an aliphatic chain (hydrophobic linear chain) with an alcohol group at one of its ends and has emulsifying properties, which is why it has been used in both, the cosmetic and food industries. Currently, docosanol 10% cream is approved for the treatment of HSV-1 lesions. However, the literature available on its effectiveness is somewhat scarce and more studies seem to be required to compare its effectiveness side by side with other compounds such as acyclovir 5% cream and Xerese^®^ (Woo and Challacombe, 2007). One study indicates that docosanol 10% cream reduces the healing time of oral lesions due to HSV-1 and HSV-2 by 18 h (Sacks et al., 2001). The mechanism of action of docosanol would be mediated by the inhibition of the fusion of the virus to the cell membrane (Pope et al., 1998).

On the other hand, another drug marketed to treat skin lesions due to HSV-2 infection is Viroxyn^®^ (Quadex Pharmaceuticals), which consists of benzalkonium chloride, a Category III antiseptic, that is also used for various applications, mainly as a biocidal preservative. This compound would act as a virucidal agent over herpes simplex viruses (Bélec et al., 2000). Although Viroxyn has been sold for more than 16 years, there are only limited studies that have evaluated its effectiveness, some proposing that it is more effective than Abreva^®^ (McCarthy et al., 2012). However, in 2016 the FDA announced a ban on the sale of numerous bactericidal ingredients, leaving benzalkonium chloride in a “stand-by” status until obtaining clinical results that prove its safety in humans, so it may eventually be recalled (“Safety and Effectiveness of Consumer Antiseptics; Topical Antimicrobial Drug Products for Over-the-Counter Human Use.” 2016-09-06. Retrieved October 05, 2016) (Wolf, 2017).

Finally, another compound marketed for treating skin lesions caused by HSV-1 is Novitra^®^, which consists of a zinc oxide-based cream. This compound has been shown to reduce HSV-1 skin lesions by up to 1.5 days, compared to untreated individuals (Godfrey et al., 2001). Other aspects, such as pain formation and itching, were also shown to be improved with its use. Table 1 summarizes the antiviral compounds discussed above.

**TABLE 1:** Approved and experimental antiviral drugs against HSV-1 and HSV-2 (non-botanical).

Drug	Mechanism of action	Type of molecule	Status	References
Acyclovir	Inhibitor of viral DNA replication	Nucleoside analog	Approved by FDA and EMA	Gnann et al., 1983
Valacyclovir	Inhibitor of viral DNA replication	Nucleoside analog	Approved by FDA and EMA	Schuster et al., 2016
Penciclovir	Inhibitor of viral DNA replication	Nucleoside analog	Approved by FDA and EMA	Meira et al., 2019
Famciclovir	Inhibitor of viral DNA replication	Nucleoside analog	Approved by FDA and EMA	Mondal, 2007
Ganciclovir	Inhibitor of viral DNA replication	Nucleoside analog	Approved by FDA and EMA	Al-Badr and Ajarim, 2018
Foscarnet	Inhibitor of viral DNA replication	Pyrophosphate analog	Approved by FDA and EMA	Crumpacker, 1992
Cidofovir	Inhibitor of viral DNA replication	Nucleotide analog	Approved by FDA and EMA	Lea and Bryson, 1996
Docosanol	Viral entry inhibitor	Saturated alcohol	Approved by FDA and EMA	Treister and Woo, 2010
Benzalkonium chloride	Virucidal	Alkylamine	Stand-by in the United States	Taylor-Robinson and Ballard, 2001
Idoxuridine	Inhibitor of viral DNA replication	Deoxyuridine analog	Approved by FDA and EMA	Chou and Hong, 2014; Wilhelmus, 2015; De Clercq and Li, 2016; Roozbahani and Hammersmith, 2018
Vidarabine	Inhibitor of viral DNA replication	Nucleoside analog	Approved by FDA and EMA	Chou and Hong, 2014
Trifluridine	Inhibitor of viral DNA replication	Nucleoside analog	Approved by FDA and EMA	Wilhelmus, 2015
Brivudine	Inhibitor of viral DNA replication	Nucleoside analog	Approved by EMA	De Clercq, 2019
Brincidofovir	Inhibitor of viral DNA replication	Nucleotide analog	Under FDA Review	Lee et al., 2018
Amenamevir	Inhibitor of viral DNA replication	Helicase-Primase inhibitor	Being assessed in clinical trials	Kawashima et al., 2017
Pritelivir	Inhibitor of viral DNA replication	Helicase-Primase inhibitor	Being assessed in clinical trials	Wald et al., 2014
Nelfinavir mesylate	Inhibits the maturation and export of viral particles	HIV-1 protease inhibitor	Being assessed in clinical trials for HSV	Kalu et al., 2014

### Other Anti-HSV Compounds

Other anti-HSV compounds, are the nucleoside analogs idoxuridine and vidarabine, which are currently discontinued as there are at present better and more effective treatments available. Trifluridine, which is also a nucleoside analogs is mainly used to treat herpetic keratitis.

Idoxuridine is a thymidine (pyrimidine) analog that was identified as the first effective topical agent against HSV infection (Chou and Hong, 2014; Wilhelmus, 2015), and was mainly used topically as an ointment for treating epithelial keratitis caused by HSV-1 infection of the corneal epithelium (Roozbahani and Hammersmith, 2018). However, its efficacy was clouded by its toxicity to the corneal epithelium of the eye and poor hydrosolubility and thus, has been currently replaced in favor of more effective, better-tolerated and less-toxic compounds (Wilhelmus, 2015).

On the other hand, vidarabine is a purine analog with fewer side-effects than idoxuridine, yet it is also poorly soluble and therefore its use is limited to topical formulations, being less preferable than other current drugs available (Chou and Hong, 2014).

Trifluridine on the other hand is a synthetic pyrimidine nucleoside that is frequently used for the treatment of herpetic keratitis as a topical formulation. This drug was approved by the FDA in 1980 for its use as a 1% solution treatment for HSV-related keratitis and is at present one of the most common used topical antiviral for this type of affection in the United States (Chou and Hong, 2014), with considerable effectivity reported (Wilhelmus, 2015). However, local side effects have been described, some particularly severe. Table 1 summarizes the antiviral activity of these compounds.

Finally, another anti-HSV agent is brivudine (BVDU), a pyrimidine analog that acts as a prodrug, phosphorylated by viral thymidine kinase only and thus targeting the viral DNA polymerase (Wilhelmus, 2015). This compound has been proven to be efficacious against HSV-1 and at least as effective as acyclovir in the treatment of HSV-1 infection (Chou and Hong, 2014). Currently, is mainly used in different countries for treatment of VZV infections (De Clercq, 2019).

### Compounds Against HSVs Currently Being Assessed in Clinical Trials

Currently, several new anti-herpetic drugs are being assessed in clinical trials, such as brincidofovir (Quenelle et al., 2010; Prichard et al., 2011), amenamevir (Chono et al., 2010), pritelivir (Wald et al., 2014), and nelfinavir mesylate (Kalu et al., 2014).

Brincidofovir is an acyclic nucleotide phosphonate, similar to cidofovir, yet it is conjugated to a lipid (Jiang et al., 2016). When brincidofovir enters the cell, the lipid sidechain is cleaved and the compound is phosphorylated, acting as a substrate inhibitor for the viral DNA polymerase. Noteworthy, brincidofovir accumulates within the cell significantly more than cidofovir, and has up to 1,000-fold higher antiviral activity as compared to the latter (Hostetler, 2009). Brincidofovir was evaluated in phase III clinical trial that has concluded, yet to our knowledge the results have not been reported (ClinicalTrials.gov Identifier: NCT01143181).

On the other hand, amenamevir and pritelivir target the viral DNA helicase/primase complex (H/P) (Kleymann et al., 2002; Poole and James, 2018). There are three finished clinical studies for pritelivir, yet similar to brincidofovir, the results have not been reported to the best of our knowledge (ClinicalTrials.gov Identifier: NCT01047540, NCT01658826, and NCT02871492). There is also one clinical study that is currently ongoing and is in the recruitment phase for immunocompromised subjects with acyclovir-resistant mucocutaneous HSV-1 or HSV-2 infection (ClinicalTrials.gov Identifier: NCT03073967). Amenamevir is an oxadiazolephenyl derivate that belongs to the helicase-primase group of inhibitors and has been evaluated in at least three clinical trials, although the results have not been published (ClinicalTrials.gov Identifier: NCT02209324, NCT01959295, and NCT02852876) (Chono et al., 2010). Regretfully, a study indicates that amenamevir displayed adverse events in an early clinical phase against HSV-1 and HSV-2 (Chono et al., 2010).

Finally, nelfinavir mesylate, the mesylate salt of the antiviral drug nelfinavir which has been characterized as a HIV-1 protease inhibitor (Patick et al., 1996; Markowitz et al., 1998), was found to have antiviral activity against HSV-1 and other herpesviruses and to inhibit the maturation and export of viral particles (Kalu et al., 2014). As a consequence, nelfinavir mesylate is currently being assessed in a clinical study for the treatment of patients with Kaposi’s sarcoma, as well as its potential effectivity activity against HSV-1 and HSV-2, which is a tertiary goal in this study (ClinicalTrials.gov Identifier: NCT03077451).

## Botanical Compounds with Antiviral Activity Against HSVs

### Algae-Derived Compounds With Antiviral Activity Against HSVs in Cell Cultures

**FIGURE 1:**
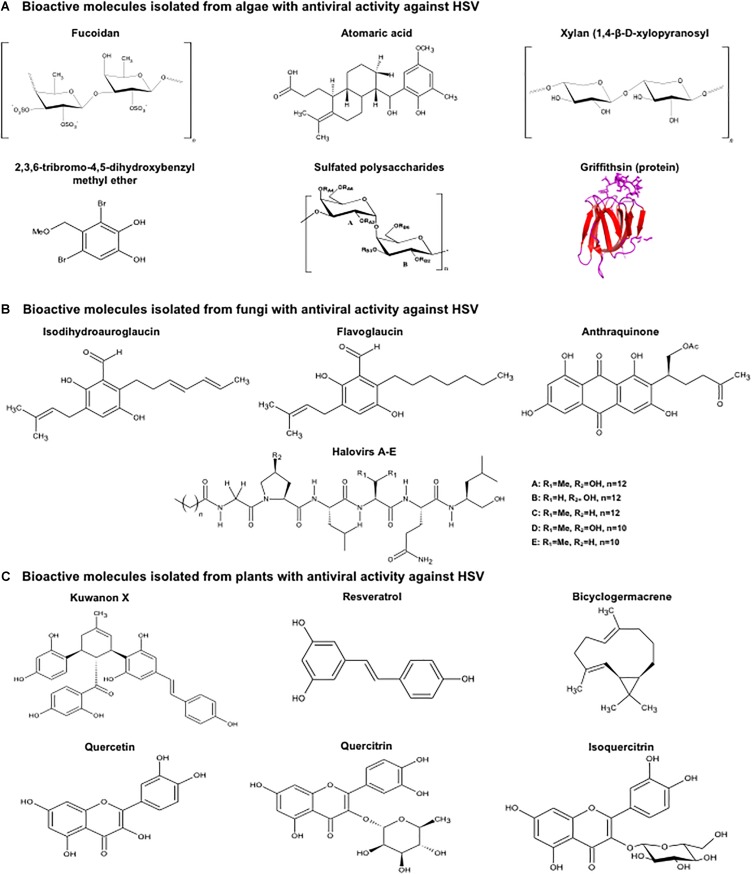
Structure of botanical molecules with antiviral activity against herpes simplex viruses. Structure of molecules derived from **(A)** algae, **(B)** fungi and **(C)** plants that have been reported to have antiviral activity against HSV-1, HSV-2 or both viruses. Molecular structures were drawn using ACD/ChemSketch^TM^, (version 2018.1.1, Advanced Chemistry Development, Inc., Toronto, ON, Canada, www.acdlabs.com, 2019). Griffithsin (Protein Data Bank accession number 2GUD) was modeled using PyMOL^TM^ (Molecular Graphics System, Version 1.3, Schrödinger, LLC).

Many studies have reported the existence of algae with bioactive compounds that display potent antiviral activity against numerous viruses, such as dengue (Lee et al., 2006; Pujol et al., 2012), avian influenza (Gerber et al., 1958), HIV (Lee et al., 1999; Thuy et al., 2015), human papillomavirus (HPV) (Buck et al., 2006), and picornavirus (Lee et al., 2009). Also, numerous studies have reported algae with antiviral activity against herpes simplex viruses (Faral-Tello et al., 2012; Ribeiro et al., 2012; Hassan et al., 2015). A study performed in Brazil analyzed more than 36 species of algae from its coasts and reported that four of them had significant antiviral activity against both, HSV-1 and HSV-2. In their study, the authors suggested that the antiviral activity of extracts of green alga *Stypopodium zonale* (Ochrophyta) against HSV-1 was related to the secondary metabolite atomaric acid (Figure 1) (Ribeiro et al., 2012). On the other hand, the antiviral activity of *Ulva fasciata* and *Codium decorticatum* against HSV-1 were shown to be mediated by fatty acids present in high concentrations in the extracts, yet the precise molecules involved were not identified or reported (Ribeiro et al., 2012). For the red alga *Laurencia dendroidea*, the antiviral activity against HSV-1 was likely mediated by sesquiterpenes (Ribeiro et al., 2012).

Another example of an alga with antiviral effects against HSV is *Hypnea musciformis*, a red seaweed present in Italy, which has shown strong antiviral activity against HSV-1, even in different aqueous fractions obtained from its processing. Several mechanisms of action for these preparations were identified, such as virucidal activity and the inhibition of virus binding into the cell (Mendes et al., 2012). Other reports have also described antiviral effects for different algae extracts against HSVs, such as *Xalas*, a derivative of *Scinaia hatei* that has antiviral activity against HSV-1 and HSV-2, which inhibits the entry of the virus into the cell, and is likely mediated by sulfated xylans present in the extract (Figure 1) (Damonte et al., 2009). On the other hand, *Osmundaria obtusiloba*, an algae obtained from the Brazilian coast was reported to have antiviral activity against both, HSV-1 and HSV-2, which was suggested to be mediated by algae glycolipids interacting with viral glycoproteins (Souza et al., 2012). *Padina pavonia*, another algae that inhibits HSV-1 replication, has been reported to have a bioactive compound consisting of sulfated polysaccharides (fucoidan) that hampers the binding of the virus to the surface of the cell (Figure 1) (Hayashi et al., 2008).

Extracts derived from the green microalga *Haematococcus pluvialis* have also shown anti-herpetic activity. Extracts obtained from this microalga through pressurized liquid extraction displayed inhibitory action against HSV-1 replication, which was suggested to be mediated by an inhibition in the attachment of the virus to the host cell, the virus-cell fusion process and/or virus entry into the cell (Santoyo et al., 2012). Another example of an alga extract with antiviral activity against HSV-1 was obtained from *Cystoseira myrica*, which strongly inhibits the replication of this virus (Zandi et al., 2007).

Interestingly, researchers have isolated and studied a chemically modified polysaccharide from the green alga *Enteromorpha compressa* with anti HSV-1 activity (Figure 1). Notably, this study reported total viral inhibition when the evaluated compound was added to human Hep-2 cells infected with a clinical isolate of HSV-1 in a time-of-addition assay. Because the effect was maintained when applied post-treatment, the authors suggested that the antiviral activity might be mediated by the inhibition of virus replication and/or viral protein synthesis (Lopes et al., 2017).

Another alga which has been studied is *Eucheuma gelatinae*, a red alga that is widespread in tropical and subtropical regions. Polysaccharides obtained from this organism were tested for their antiviral activity against HSV *in vitro* using Vero cells infected with ACV-sensitive or ACV-resistant HSV-1 and HSV-2 isolates (Figure 1). In this study, strong antiviral activity against HSV was observed in early stages of infection affecting the attachment of HSV. Additionally, it was shown experimentally that viral protein synthesis was affected through the evaluation of the expression of the viral protein VP5, as well as the cellular localization of this protein which is normally found in the nucleus. After the treatment with the alga extract, VP5 was mainly found in the cytoplasm (Jin et al., 2015). Table 2 summarizes the antiviral activity of these compounds.

**TABLE 2:** Botanical compounds and extracts with antiviral activity against HSV-1 and HSV-2.

Species (common name)	Mechanism of action	Virus assessed	Assessed in cell cultures	Assessed in animal models	Active molecule	References
**Algae**						
*Hypnea musciformis* (Hooked Weed)	Virucidal, inhibition of viral binding, inhibition of viral entry	HSV-1	✓	Unknown or not reported	Unknown or not reported	Mendes et al., 2012
*Stypopodium zonale* (*Ochrophyta*)	Unknown or not reported	HSV-1	✓	Unknown or not reported	Atomaric acid	Ribeiro et al., 2012
*Scinaia hatei*	Inhibition of viral entry	HSV-1 and HSV-2	✓	Unknown or not reported	Xylan (1,4-β-D-xylopyranosyl)	Damonte et al., 2009
*Osmundaria obtusiloba*	Interaction with viral glycoproteins	HSV-1 and HSV-2	✓	Unknown or not reported	Sulfoquinovosyldia cylglycerol	Souza et al., 2012
*Padina pavonica* (Peacock’s Tail)	Inhibition of viral binding	HSV-1	✓	✓	Fucoidan	Hayashi et al., 2008
*Haematococcus pluvialis*	Inhibition of viral binding, inhibition of viral entry	HSV-1	✓	Unknown or not reported	Unknown or not reported	Santoyo et al., 2012
*Cystoseira myrica* (False Sargassum)	Viral inhibition before and after absorption	HSV-1	✓	Unknown or not reported	Unknown or not reported	Zandi et al., 2007
*Enteromorpha compressa*	Inhibition of viral replication, inhibition of viral protein synthesis	HSV-1	✓	Unknown or not reported	Sulfated polysaccharides	Lopes et al., 2017
*Eucheuma gelatinae* (Guso)	Virucidal, inhibition of viral binding, inhibition of viral gene expression	HSV-1	✓	Unknown or not reported	Sulfated polysaccharides	Jin et al., 2015
*Griffithsia* sp. (*Griffiths’s Coral Weed*)	Inhibition of viral binding, inhibition of viral entry	HSV-2	✓	✓	Griffithsin	Nixon et al., 2013; Levendosky et al., 2015; Derby et al., 2018
*Symphyocladia latiuscula*	Virucidal	HSV-1	✓	✓	2,3,6-tribromo-4,5-dihydroxybenzyl methyl ether	Park et al., 2014
*Undaria pinnatifida* (Wakame)	Inhibition of viral binding	HSV-1 and HSV-2	✓	✓	Fucoidan	Hayashi et al., 2008; Lee et al., 2004
**Fungi**						
*Aspergillus ruber*	Inhibition of viral binding, inhibition of entry	HSV-1	✓	Unknown or not reported	Isodihydroauroglaucin Flavoglaucin	Liang et al., 2018
*Aspergillus versicolor*	Unknown or not reported	HSV-1	✓	Unknown or not reported	Anthraquinones	Huang et al., 2017
*Scytalidium*	Virucidal	HSV-1 and HSV-2	✓	Unknown or not reported	Halovirs A – E	Rowley et al., 2003
*Inonotus obliquus*	Inhibition of viral binding, inhibition of viral entry	HSV-1	✓	Unknown or not reported	aqueous extract from *Inonotus obliquus* (AEIO)	Pradeep et al., 2019
*Ganoderma lucidum* (Lingzhi Mushroom)	Inhibition of viral binding, inhibition of viral entry	HSV-1 and HSV-2	✓	Unknown or not reported	Polysaccharides	Eo et al., 2000
*Agaricus brasiliensis* (Almond Mushroom)	Inhibition of viral binding, inhibition of viral entry	HSV-1 and HSV-2	Unknown or not reported	✓	Polysaccharides	Cardozo et al., 2013
*Grifola frondosa* (Maltake)	Virucidal	HSV-1	✓	✓	Protein	Gu et al., 2007
*Rozites caperata*	Unknown or not reported	HSV-1	✓	✓	Peptides	Pradeep et al., 2019
**Plants**						
*Peganum harmala* (Wild Rue)	Inhibition of viral entry	HSV-2	✓	Unknown or not reported	Harmine	Benzekri et al., 2018
*Melia azedarach* (*Chinaberry Tree*)	Unknown or not reported	HSV-1 and HSV-2	✓	✓	Meliacine (Glycopeptide)	Petrera and Coto, 2009; Barquero et al., 1997
*Labiatae*	Virucidal, inhibition of viral binding, inhibition of viral entry	HSV-1 and HSV-2	✓	Unknown or not reported	Unknown or not reported	Brand et al., 2016
*Verbenaceae*	Virucidal, inhibition of viral binding, inhibition of viral entry	HSV-1 and HSV-2	✓	Unknown or not reported	Unknown or not reported	Brand et al., 2016
*Glechon spathulata*	Inhibition after viral attachment	HSV-1	✓	Unknown or not reported	Bicyclogermacrene	Venturi et al., 2015
*Glechon marifolia*	Inhibition after viral attachment	HSV-1	✓	Unknown or not reported	Bicyclogermacrene	Venturi et al., 2015
*Aglaia odorata* (Chinese Perfume Plant)	Inhibition after viral attachment	HSV-1	Unknown or not reported	✓	Unknown or not reported	Lipipun et al., 2003
*Moringa oleifera* (Moringa)	Inhibition after viral attachment	HSV-1	Unknown or not reported	✓	Unknown or not reported	Lipipun et al., 2003
*Ventilago denticulata*	Inhibition after viral attachment	HSV-1	Unknown or not reported	✓	Unknown or not reported	Lipipun et al., 2003
*Morus alba* (White Mulberry)	Inhibition of early stages of viral infection	HSV-1 and HSV-2	✓	Unknown or not reported	Kuwanon X	Ma et al., 2016
*Houttuynia cordata* (Fish Mint)	Inhibition of NF-κB activation, inhibition of viral binding, inhibition of viral entry	HSV-1 and HSV-2	✓	Unknown or not reported	Quercitin, Isoquercitrin and Quercitrin	Chen et al., 2011 and Hung et al., 2015
*Veratum grandiflorum*	Inhibition of viral replication	HSV-1 and HSV-2	✓	Unknown or not reported	Resveratrol	Faith et al., 2006; Leyton et al., 2015
*Eucalyptus camaldulensis* (River Red Gum)	Inhibition before and after virus adsorption	HSV-1 and HSV-2	✓	Unknown or not reported	Unknown or not reported	Mahmoud, 2017
*Eucalyptus sideroxylon* (Mugga)	Virucidal, inhibition of viral entry, post-infection antiviral effects	HSV-1 and HSV-2	✓	Unknown or not reported	Unknown or not reported	Okba et al., 2017
*Eucalyptus globulus* (Southern Blue Gum)	Unknown or not reported	HSV-1 and HSV-2	✓	Unknown or not reported	Tereticornate A. Cypellocarpin C	Brezáni et al., 2018
*Cassia stipulacea* (Quebracho)	Inhibition after virus adsorption	HSV-1	✓	Unknown or not reported	Unknown or not reported	Pacheco et al., 1993
*Escallonia illinita* (Ñipa)	Inhibition after virus adsorption	HSV-1	✓	Unknown or not reported	Unknown or not reported	Pacheco et al., 1993
*Aristotelia chilensis* (Chilean Wineberry)	Inhibition after virus adsorption	HSV-2	✓	Unknown or not reported	Unknown or not reported	Pacheco et al., 1993
*Drymis winteri* (Winter’s Bark)	Inhibition after virus adsorption	HSV-2	✓	Unknown or not reported	Unknown or not reported	Pacheco et al., 1993
*Elytropus chilensis* (Quilmay)	Inhibition after virus adsorption	HSV-2	✓	Unknown or not reported	Unknown or not reported	Pacheco et al., 1993
*Luma apiculata* (Chilean Myrtle)	Inhibition after virus adsorption	HSV-2	✓	Unknown or not reported	Unknown or not reported	Pacheco et al., 1993
*Quillaja saponaria* (Soap Bark Tree)	Virucidal, inhibition of virus binding, inhibition of virus entry	HSV-1	✓	Unknown or not reported	Unknown or not reported	Roner et al., 2007
*Melaleuca alternifolia* (Tea Tree)	Inhibition of virus binding, inhibition of virus entry	HSV-1 and HSV-2	✓	Unknown or not reported	Unknown or not reported	Garozzo et al., 2009
*Melissa officinalis* (*Balm Mint*)	Unknown or not reported	HSV-2	✓	Unknown or not reported	Unknown or not reported	Allahverdiyev et al., 2004
*Alpinia officinarum* (*Lesser Galangal*)	Inhibition after virus adsorption	HSV-1	✓	✓	Unknown or not reported	Kurokawa et al., 1995
*Geum japonicum* (*Asian Herb Bennet*)	Inhibition after virus adsorption	HSV-1	✓	✓	Unknown or not reported	Kurokawa et al., 1995
*Paeonia suffruticosa* (*Mudan*)	Inhibition after virus adsorption	HSV-1	✓	✓	Unknown or not reported	Kurokawa et al., 1995
*Phellodendron amurense* (*Amur Cork Tree*)	Inhibition after virus adsorption	HSV-1	✓	✓	Unknown or not reported	Kurokawa et al., 1995
*Polygala tenuifolia* (*Yuan Zhi*)	Inhibition after virus adsorption	HSV-1	✓	✓	Unknown or not reported	Kurokawa et al., 1995
*Polygonum cuspidatum* (*Asian Knotweed*)	Inhibition after virus adsorption	HSV-1	✓	✓	Unknown or not reported	Kurokawa et al., 1995
*Rhus javanica* (*Java Brucea*)	Inhibition after virus adsorption	HSV-1	✓	✓	Unknown or not reported	Kurokawa et al., 1995
*Syzygium aromaticum* (Clove)	Inhibition after virus adsorption	HSV-1	✓	✓	Unknown or not reported	Kurokawa et al., 1995
*Terminalia arjuna* (Arjun Tree)	Inhibition after virus adsorption	HSV-1	✓	✓	Unknown or not reported	Kurokawa et al., 1995
*Terminalia chebula* (Black Myrobalan)	Inhibition after virus adsorption	HSV-1	✓	✓	Unknown or not reported	Kurokawa et al., 1995
*Alternanthera philoxeroides* (Alligator Weed)	Virucidal	HSV-1 and HSV-2	✓	✓	Chikusetsusaponin IV	Rattanathongkom et al., 2009

### Algae-Derived Compounds With Antiviral Activity Against HSVs in Animal Models

Griffithsin is a lectin extracted from the red alga *Griffithsia* sp., which has been shown to be capable of inhibiting HSV infection in a murine model of HSV-2 infection (Figure 1) (Nixon et al., 2013). The mechanism of action of Griffithsin consists on its binding to mannose N-glycosylations that blocks the infection process of HSV-2 and inhibits cell-to-cell spread of the virus. The results with this compound have been promising and its effectiveness is expected to be evaluated in humans in the near future. Additionally, Griffithsin has been evaluated as an antiviral in combination with carrageenan both, in cell cultures and in animals to evaluate the potential synergic effect of the two compounds against HSV-2 in a murine model of infection. The study showed a strong reduction in HSV-2 infection when applied together as a prophylactic, namely between 10 min and 1 h prior to infection (Levendosky et al., 2015). A more recent article reported the efficacy of Griffithsin in combination with carrageenan against HSV-2 infections together with HPV and HIV-1, which was evaluated in both, a murine and rhesus macaque model using a vaginal fast-dissolving insert (Derby et al., 2018). This study showed that a fast-dissolving vaginal insert with low moisture content is able to protect against SHIV in macaques, while in mice it showed promising results protecting against HSV-2 and HPV.

Notably, *Symphyocladia latiuscula* is a red macroalga that has been reported to produce compounds with virucidal antiviral activity against HSV-1 (Figure 1). Vero cells infected with HSV-1 and treated with extracts from this microalga showed reduced plaque formation. The antiviral effect of these compounds has also been evaluated in a murine model skin infection, which showed decreased skin lesions compared to controls when administrated 4 h before infection and then three times per day for 6–10 days. Also, skin obtained from these treated and infected animals showed lesser plaque forming units than the control (Park et al., 2014).

On the other hand, Hayashi and colleagues reported that fucoidan from the brown macroalga *Undaria pinnatifida*, which are sulfated polysaccharide, have antiviral activity against HSV-1 and HSV-2 that is mediated by hampering the binding of the virus to the cell surface (Figure 1) (Lee et al., 2004). When the effect of fucoidan was tested in another study against corneal infection with HSV-1, a reduction in herpetic lesions was found in those animals that received pretreatments with fucoidan during 1 week (Hayashi et al., 2008). Finally, a study performed by Alboofetileh et al. (2019), showed that fucoidans extracted from the brown seaweed *Nizamuddinia zanardinii* exert strong antiviral activity against HSV-2 infection. They found that an algal extract containing this compound inhibited the attachment of HSV-2 to Vero cells, inhibiting the early phase of HSV-2 infection (Alboofetileh et al., 2019). Table 2 summarizes the antiviral activity of these compounds.

Another study reported that chemically modified polysaccharides from the green algae *E. compressa* of the *Ulvaceae* family, has antiviral activity against HSV-1 infection. In this work the green algae was processed and chemically modified polysaccharides were purified and tested in plaque reduction assays, determining an antiviral effect mediated after virus penetration (Lopes et al., 2017).

Interestingly, a recent article showed antiviral activity against HSV-2 for two purified sulfated polysaccharides isolated from the brown alga *Sargassum henslowianum*, a species found in southeastern China and Asia (Sun et al., 2019). In this work, algae extracts were treated to purify the polysaccharides named SHAP-1 and SHAP-2, which were later evaluated in viral plaque formation assay for both HSV-1 and HSV-2 virus. However, following experiments in this research article focused on HSV-2 rather than HSV-1, arguing greater clinical value for the former (Sun et al., 2019). Time-of-addition assays, as well and adsorption and penetration assays suggest an antiviral effect at early stages of infection (Sun et al., 2019).

### Fungus-Derived Compounds With Antiviral Activity Against HSVs in Cell Cultures

Fungus-derived compounds have also been explored for identifying novel molecules with antiviral activity against HSV-1 and HSV-2. The fungus *Aspergillus versicolor* has been shown to produce secondary metabolites, such as anthraquinones with anti-herpetic activity (Figure 1). In the study by Huang et al. (2017), three anthraquinones were found to have antiviral effects against HSV-1 *in vitro* using Vero cells. Another fungus of the *Aspergillus* genus showed antiviral activity against HSV-1, with two secondary metabolites flavoglaucin and isodihydroauroglaucin derived from *A. ruber* being assessed, and two compounds showing anti-HSV-1 effects (Figure 1) (Liang et al., 2018). In another study, peptides produced by the marine-derived fungus *Scytalidium* were found to have antiviral activity against HSV-1 and HSV-2, particularly by a peptide named Halovir, which was suggested to have virucidal activity when in direct contact with HSV-1 and HSV-2 (Figure 1) (Rowley et al., 2003).

Furthermore, an aqueous extract from *Inonotus obliquus* (AEIO) was shown to inhibit HSV-1 infection in Vero cells (Pradeep et al., 2019). the antiviral activity was detected at early times during viral infection, suggesting AEIO blocks viral entry, particularly membrane fusion (Pradeep et al., 2019).

Interestingly, fungus proteins that inhibit HSV infection have also been identified. Two proteins that bind polysaccharides from the mushroom *Ganoderma lucidum* were found to have antiviral effects against HSV-1 and HSV-2. One was named the neutral protein bound to polysaccharide (NPBP) and the other the acidic protein bound to polysaccharide (APBP). Although APBP had more potent antiviral activity than NPBP, both inhibited plaque formation by both types of HSV. Interestingly, it was found that the mechanism of action of APBP was mediated by the inhibition of the attachment and penetration of the virus into Vero cells (Eo et al., 2000). Table 2 summarizes the antiviral activity of these compounds.

### Fungus-Derived Compounds With Antiviral Activity Against HSVs in Animal Models

Sulfated compounds isolated from a polysaccharide (MI-S) derived from *Agaricus brasiliensis* have shown antiherpetic activity against HSV-1 and HSV-2 (De Sousa Cardozo et al., 2014). MI-S inhibited viral adsorption and penetration into Vero cells and had a synergistic effect with acyclovir (De Sousa Cardozo et al., 2014). Interestingly, this compound was also shown to reduce the severity of HSV-2 disease in a murine genital infection model with one single application (Cardozo et al., 2013).

On the other hand, Qing et al., reported that a protein from *Grifola frondosa* (GFAHP) had antiviral activity against HSV-1, which was shown to have virucidal effects in cell cultures and suppressed viral entry into Vero cells (Gu et al., 2007). Furthermore, GFAHP also showed antiviral activity against HSV-1 in animals when applied topically to the cornea of mice. Mice treated with GFAHP had a significant reduction in blepharitis, vascularization and stromal disease, as well as reduced viral replication in the cornea (Gu et al., 2007). Notably, the antiviral protein RC28 obtained from the fungus *Rozites caperata* showed antiviral activity against HSV-1 in Vero cells (Pradeep et al., 2019). Moreover, the authors evaluated the antiviral effect of RC28 in an animal model and observed that this peptide decreased the severity of stromal keratitis (Pradeep et al., 2019). Table 2 summarizes the antiviral activity of these compounds.

### Plant-Derived Compounds With Antiviral Activity Against HSVs in Cell Cultures

Plant extracts have received particular attention when searching for new molecules with anti-herpetic activity (Li et al., 2017; Akram et al., 2018). Interestingly, numerous plant-derived extracts and compounds have been reported to inhibit HSV replication. For instance, organic extracts belonging to the *Peganum harmala* species have been described to have antiviral activity against HSV-2 and to interfere with virus entry (Benzekri et al., 2018).

Essential oils extracted from plants belonging to the *Labiatae* and *Verbenaceae* families have also been shown to have antiviral activity against HSV. Vero cells incubated with HSV and plant-extracted essential oils for 48–72 h significantly reduced HSV-1 and HSV-2 viral titers. Interestingly, their mechanisms of action were found to be related to the pre-infective stages (Brand et al., 2016). Another study that also assessed essentials oils, but extracted from *Glechon spathulata* and *Glechon marifolia* identified antiviral activity against HSV-1, which was effective after the infection of Vero cells (Figure 1) (Venturi et al., 2015). Eucalyptus essential oils are used to treat symptoms during cough and bronchitis, in numerous presentations, such as ointments, liniments, in oral form and in vapor baths as inhalants (Kaur et al., 2013). The main components of the essential oils from the Eucalyptus species are cineole and alpha-pinene (Sebei et al., 2015). One study evaluated the Australian tea tree oil and Eucalyptus oil over HSV and reported antiviral activity against both, HSV-1 and HSV-2. Treatments before, during or post-infection determined that these compounds had virucidal activity affecting the virus before or during adsorption, before the virus had entered the cells (Schnitzler et al., 2001). Ethanolic extracts from the leaves of *E. camaldulensis* were shown to inhibit HSV-1 and HSV-2 infection when the extracts were added to Vero cells during- and post-infection. Additionally, in this study a synergistic effect between acyclovir and the ethanolic extracts was reported in cell cultures (Abu-Jafar and Mahmoud, 2017). In another study, researchers reported 24 new metabolites in the leaves of *E. sideroxylon* and four new metabolites within the genus *Eucalyptus* that have antiviral activity using an ultra-performance liquid chromatography coupled to photodiode-array and electrospray ionization mass spectrometer (UPLC/PDA/ESI-qTOF-MS). Within the antiviral activities detected, there were compounds that also inhibited hepatitis A, coxsackie and adenoviruses, besides HSV-1 and HSV-2. Interestingly, the highest antiviral activity was observed against HSV-2, with the antiviral effect acting pre-treatment (virucidal), thus inhibiting virus entry and subsequent infection processes, while the antiviral effect against HSV-1 was only observed when the extract was incubated with the virus previous to the cell infection (Okba et al., 2017). In addition, twelve compounds isolated from the leaves and twigs of *E. globulus* were found to have antiviral activity against HSV-1 and HSV-2. In this study, Tereticornate A was identified to have the greatest activity against HSV-1, which was higher than acyclovir. Cypellocarpin C displayed the strongest antiviral activity against HSV-2, greater than that observed for acyclovir (Brezáni et al., 2018).

Other essential oils, particularly those obtained from the leaves of *Melissa officinalis*, which is better known as lemon balm, have also been shown to have antiviral activity against both, HSV-1 and HSV-2 with the antiviral activity attributed to tannins and non-tannin polyphenolic fractions within the extract (Mazzanti et al., 2008). Another study determined that volatile oils from *M. officinalis* Lamiaceae had antiviral activity against HSV-2 (Allahverdiyev et al., 2004). On the other hand, extracts from *Aglaia odorata*, *Moringa oleifera*, and *Ventilago denticulate* have also been shown to have antiviral activity against wild-type and drug-resistant HSV-1 isolates, yet the mechanisms of action seems to not have been identified yet, or reported (Lipipun et al., 2003).

Plants used in traditional Chinese medicine have also been tested and found to have antiviral activity (Ganjhu et al., 2015). Recently, a study reported that leaves from Mulberry (*Morus alba* L.), a plant that is common in Asia, has antiviral properties against HSV-1 and HSV-2. The active compound within this plant has been reported to be Kuwanon X, a stilbene polyphenol derivative, which has antiviral activity over HSV at multiple steps of the infection process, inhibiting cellular adsorption and penetration, as well as the expression of HSV-1 immediate early and late genes, and the synthesis of HSV-1 DNA (Figure 1) (Ma et al., 2016). Another study showed that aqueous extracts from *Houttuynia cordata*, a Chinese herbal medicine, blocks HSV-2 infection by inhibiting NF-κB activation, a host transcription factor that has been reported to be required for effective HSV infection (Amici et al., 2006). In order to identify the compounds with antiviral activity within *H. cordata*, several flavonoid compounds in this plant were evaluated individually to determine their capacity to block the replication cycle of HSV-2. Quercetin, quercitrin, and isoquercitrin, the major flavonoid compounds found within *H. cordata* were found to be strong inhibitors of HSV-2 activity (Figure 1) (Chen et al., 2011). A subsequent study from another group determined that the mechanism of action behind the anti-herpetic activity of *H. cordata* occurred at multiple levels, such as at the adsorption level, entry, post-infection acting over NF-κB and had virucidal activity (Hung et al., 2015). Another stilbene compound that inhibits NF-κB activation is resveratrol, which was isolated from *Veratum grandiflorum* (Chen et al., 2012), and it is the main bioactive compound found in berries, peanuts, legumes and other plant-derived matrices, as well as red wine. Several studies have reported antiviral properties for this compound over the replication cycles of ACV-resistant and wild-type HSV-1 and HSV-2, both in cell cultures and in animal models (Figure 1) (Faith et al., 2006; Leyton et al., 2015).

Aqueous and hydroalcoholic extracts derived from native plants of Chile have also been investigated for their antiviral activity against HSV-1 and HSV-2. Hydroalcoholic extracts of *Cassia stipulacea* and *Escallonia illinita* displayed antiviral activity against HSV-1, while hydroalcoholic extracts from *Aristotelia chilensis*, *Drymis winteri*, *Elytropus chilensis*, as well as an aqueous extract from *Luma apiculata* showed antiviral activity against HSV-2. The active antiviral compounds within these preparations have not been identified or reported yet (Pacheco et al., 1993). In addition, another group determined that aqueous extracts from *Quillaja saponaria*, a Chilean soapbark tree that is endemic in the central zone of Chile, has antiviral activity against HSV-1 and other viruses. This extract, which is currently used in food and beverages was reported to have virucidal activity by blocking the attachment of viruses to the cell surface (Roner et al., 2007). Table 2 summarizes the antiviral activity of these compounds.

### Plant-Derived Compounds With Antiviral Activity Against HSVs in Animal Models

Several studies have assessed the antiviral effects of plant-derived compounds against herpes simplex virus in animal models either, alone or combined with acyclovir. One of these studies found that each of the following, *Geum japonicum* Thunb., *Rhus javanica* L., *Syzygium aromaticum* (L.), or *Terminalia chebula Retzus* displayed increased antiviral activity against HSV-1 when combined with acyclovir, as compared to acyclovir alone (Kurokawa et al., 1995). On the other hand, extracts from *A. odorata*, *M. oleifera*, and *V. denticulate* were shown to have antiviral effects against HSV-1 upon cutaneous infections in BALB/c mice. Here, the plant extracts combined with ACV and orally administered to the mice were shown to hamper the development and progression of HSV-1 skin lesions and increased the mean survival times of the animals (Lipipun et al., 2003). Chikusetsusaponin IV, a compound extracted from *Alternanthera philoxeroides* has shown to have antiviral activity against HSV-2 when the compound is added to the inoculum for 1 h before viral infection or immediately after viral infection (Rattanathongkom et al., 2009). The authors suggested that the mechanism of action of the antiviral activity action of Chikusetsusaponin IV was virucidal (Rattanathongkom et al., 2009). Moreover, Chikusetsusaponin IV showed antiviral activity against HSV-2 genital infection in mice when administrated three times per day three days before infection and up to 7 days after infection (Rattanathongkom et al., 2009). Finally, Meliacine (MA) a glycopeptide obtained from *Melia azedarach* has been reported to have antiviral activity against acyclovir-sensitive and acyclovir-resistant HSV-1 (Barquero et al., 1997). Furthermore, MA showed favorable results against HSV-2 in a mouse model of infection when applied topically immediately after infection with HSV-2 (Petrera and Coto, 2009). Table 2 summarizes the antiviral activity of these compounds.

## Clinical Trials

Different natural compounds have been tested in clinical trials following studies performed in animal models, in such a way to validate potential new drugs for the treatment of herpes simplex virus manifestations. Regarding *herpes labialis*, a Neem tree-based cream (*Azadirachta indica*) called TheraNeem Lip Therapy that has several botanical extracts, including those obtained from organic Neem Oil, organic Coconut Oil, organic Beeswax, organic Jojoba Oil, shea Butter, sesame Oil, essential Oil of Peppermint, Vitamin E (Tocopherol) was reported to having been tested in a clinical trial (Clinicaltrials.gov Identifier NCT00985335). Regretfully, the results of this study have not been published to the best of our knowledge.

Another study involving the treatment of *herpes labialis* lesions was performed with Huanglian-Jiedu Decoction, a Chinese medicine formulation that includes four kinds of Chinese herbs; Huanglian (*Rhizoma coptidis*), Huangbo (*Cortex Phellodendri*), Huangqin (*Scutellaria baicalensis*) and Zhizi (*Scutellaria baicalensis*) (Clinicaltrials.gov Identifier NCT03469232). However, similar to the clinical study reported above, the results of this trial have not been published to date.

Additionally, a randomized controlled open-label superiority trial for herpes simplex virus labial episodes was recently performed with medical-grade kanuka honey (Semprini et al., 2019). Here, the botanical compound and acyclovir were applied topically as a cream up to five times a day and concluded that the medical-grade kanuka honey did not provide better efficacy than acyclovir 5% (Semprini et al., 2019).

On the other hand, a clinical study tested VIBLOCK, which is reportedly 100% formulated with natural products, although the composition of this cream was not described (Clinicaltrials.gov Identifier NCT03080961). The formulation tested the capacity of the formulated cream to prevent HSV-2 infection. However, the results of this study have not been reported.

## Concluding Remarks

Taken together, numerous botanical compounds derived from algae, fungi and plants have been reported to have strong antiviral activities both, in cell culture assays and in animal studies against herpes simplex viruses (HSV-1 and HSV-2). Interestingly, several mechanisms of action for these compounds have been identified, among which the most frequent seems to be virucidal activity. Other reported activities are inhibition of virus-entry into the target cells, inhibition of viral protein expression and interference with viral DNA replication, which are all essential processes for generating novel infectious viral particles (Ibáñez et al., 2017).

For botanical extracts in which the active compounds against HSV-1 and HSV-2 have not been purified or identified, their clinical application seems most likely oriented toward topical treatments for cutaneous or mucosal manifestations elicited by these viruses, although some have been shown to be effective if taken orally.

Importantly, botanical drugs derived from botanical extracts and compounds can undergo special health-regulatory conditions that favor their progress into the clinic in countries such as the United States, as botanical drugs that are marketed in this country as a dietary supplements may move forward into clinical studies without the need of non-clinical pharmacological/toxicological testing if they have already been proven to have a general recognition of safety (Botanical Drug Development Guidance for Industry, December 2016, Pharmaceutical Quality/CMC). Indeed, some botanical drug products may not require typical Phase 1 tolerability studies if the sponsors can provide adequate justification for the relevance of the prior use in humans and may eventually even be commercialized over-the-counter (OTC). Hence, such indications may significantly help botanical drugs rapidly reach the clinic and help treat both ACV-sensitive and antiviral-resistant HSV isolates. Taken together, botanical compounds have the advantage, over other synthetic drugs that they are generally recognized as safe, beneficial and are readily available resources, thus reducing the pricy steps needed for new drug discovery (Pan et al., 2013; Thomford et al., 2018). Furthermore, botanical-based remedies may be low-cost alternatives for unprivileged nations, if the botanicals are available, where access to modern medicine is difficult (Shaikh and Hatcher, 2005; Karunamoorthi et al., 2013). Botanical drugs also have the advantage, over standard small molecule drugs, that they have multiple bioactive compounds that may act synergistically to hamper virus replication, while avoiding antiviral resistance (Yuan et al., 2016; Chugh et al., 2018).

However, botanical drug interactions with other drugs have been reported and undesirable side effects may occur (Tachjian et al., 2010; Ekor, 2014; Lee et al., 2016; Borse et al., 2019). At present, the World Health Organization has developed guidelines to reinforce safety monitoring of botanical medicines using pharmacovigilance systems, as well as encouraging quality controls during production by using modern manufacture techniques and applying good manufacturing practices (WHO, 2000, 2004).

Finally, the expansion of the botanical drug market has attracted significant interest from pharmaceutical companies, which have intensified their development of pre-clinical and pharmacological studies on such drugs forecasting a 6.1% CAGR growth of approximately 31.6 billion dollars for this field in the period between 2017 and 2022 (Market Watch, 2017). Hence, a steady increase in botanical drugs reaching the market should be expected in the years to come.

## Author Contributions

All authors wrote the manuscript, designed the tables, and reviewed the manuscript.

## Conflict of Interest

The authors declare that the research was conducted in the absence of any commercial or financial relationships that could be construed as a potential conflict of interest.

## Footnotes

1 http://www.who.int/mediacentre/news/releases/2015/herpes/en/
